# Quantifying wing wear in bumble bees using fractal geometry

**DOI:** 10.1371/journal.pone.0350743

**Published:** 2026-08-19

**Authors:** Ivan Nirmalaraj, Nicholas Sookhan, J. Scott MacIvor

**Affiliations:** Department of Biological Sciences, University of Toronto Scarborough, Toronto, Ontario, Canada; Southeastern Louisiana University, UNITED STATES OF AMERICA

## Abstract

Wing wear in bees (Hymenoptera: Apidae) accumulates through flight activity and can serve as a proxy for foraging effort and age, yet methods for quantifying wear rely on coarse categorical scoring or simple geometric measurements that do not capture the complexity of wing edge degradation. We evaluate fractal dimension (FD) analysis as a quantitative morphometric for assessing wing wear and evaluate its relationship with visual scoring and body size. Unlike linear wing metrics, FD can capture subtle irregularities, yet no study has used FD to quantify wing wear in insects. We use forewing images collected from 263 workers of three North American bumble bee species (*Bombus impatiens, B. bimaculatus,* and *B. rufocinctus*) across meadows in Rouge National Urban Park (Toronto, Canada) to compare FD to traditional measures of wing condition, including wing wear scores and perimeter–area ratio. We found that FD of undamaged wings varied between species indicating interspecific variation in wing morphology. Furthermore, damaged wings correlated more strongly with visual wear scores than perimeter–area ratio, and unlike perimeter-area ratio, was not confounded by body size. Our findings highlight FD as a sensitive quantitative morphometric for wing wear and a promising tool for measurement of bee body condition, age, and foraging effort.

## Introduction

Wing wear occurs in many insect groups and is an irreversible, cumulative process that results from flight activity, collisions with vegetation, environmental exposure, and predator evasion [1]. In bees (Hymenoptera: Apidae), wing wear has been used as a proxy for foraging age [2–4] and to measure foraging effort [5,6]. Previous studies have quantified wing wear in bees using visual categorical scoring methods such as the method developed by Mueller & Wolf-Mueller [4], which uses a scale of qualitative observations of wing degradation that correlate to an increasing wing wear score (referred to here as MWM score). Others have measured loss of wing area from photographs [1,7]. While these methods have provided valuable insights, categorical scoring lacks precision and sensitivity to subtle variation, and simple area measurements do not capture the complexity of wing edge degradation.

A more precise, quantitative approach may be possible using fractal geometry. Fractal dimension (FD) analysis is a mathematical measure used to quantify the complexity of geometric structures and has been used in a range of applications such as characterizing littoral zone complexity [8], rock surface topography [9], Rorschach inkblots [10], and the shape of the flight paths of honey bees (*Apis mellifera*, Hymenoptera: Apidae) [11]. One study successfully applied FD analysis to characterize butterfly wing patterns and to distinguish species based on wing complexity [12]. Unlike linear wing metrics, FD can capture subtle irregularities, making it a potentially useful tool for assessing insect wing condition. However, no study has used FD to quantify wing wear in insects. Given its ability to capture fine structural changes, FD analysis could offer a more objective method for quantifying wing wear. In this study, we develop and apply this approach to bumble bees *(Bombus,* Hymenoptera: Apidae).

Bumble bees form colonies, and body size exhibits substantial variation among individual workers which can influence foraging through a variety of factors. Larger workers generally have greater foraging ranges, can carry larger pollen and nectar loads, and may access flowers with deeper corollas more efficiently [13–15]. However, smaller workers may experience higher wing loading and greater wingbeat frequencies, potentially leading to different patterns of wing wear accumulation [16]. Understanding how body size interacts with wing wear is important for interpreting FD as a proxy, as any size-dependent pattern could confound interpretations.

The objective of this study is to evaluate whether FD is a reliable quantitative morphometric for assessing wing wear in bumble bees by testing its correlation with the established MWM visual ordinal scoring methods and its relationship to body size. Specifically, we test whether FD differs interspecifically in *Bombus impatiens, Bombus bimaculatus* (both in the subgenus *Pyrobombus*) and *Bombus rufocinctus* (*Cullumanobombus*) workers. We test whether larger-bodied workers accrue more wing wear, and we predict that intertegular distance (ITD) will be positively correlated with wing wear metrics, including MWM wing score, perimeter-area ratio, and fractal dimension.

## Methods

### Bumble bee surveys

Seven sites were sampled within the Rouge National Urban Park (RNUP), Toronto, Ontario, Canada (S1 Fig). Bumble bee surveys were completed during the summers of 2016 and 2017. Each vegetation survey site was surveyed for bumble bees once a week beginning on May 17^th^ and ending on September 3^rd^ in 2016, and May 18^th^ to September 21^st^ in 2017. Sites were surveyed using two sampling methods. Sweep netting was completed with hand nets between 8:30–15:30 on non-windy days. Surveys were completed by either two surveyors for 15 min or a single surveyor for 30 min. Surveyors completed haphazard transect walks during which pollinating bumble bees were captured. Captured insects were placed in scintillation vials and then euthanized and stored in a −21ºC freezer at the end of the collection day. Bees were then pinned, identified to species, then curated in the Biodiversity of Urban Green Spaces (‘BUGS’) lab at the University of Toronto Scarborough.

### Specimen processing

To ensure consistency in assessing wing wear and body size, images of bumble bee worker forewings were captured at standardized dimensions and lighting conditions, and individuals’ intertegular distance (ITD) was measured using the OMAX A35180U3 high-resolution (18 megapixels) digital microscope camera. Full-resolution images (4912 x 3684 pixels) were resized to 1024 x 768 pixels.

The ITD of each individual worker was calibrated and measured using the ToupLite software in combination with the microscope camera. The wing images were also cropped for standardization at the second cubital cross vein. Using LabelMe (https://labelme.io/), the wing was manually segmented and then converted to a binary mask for subsequent analysis using Python 3.13 [17].

### Measuring wing morphometrics

The wing wear of each imaged wing was visually assessed using the MWM wing score method developed by Mueller & Wolf-Mueller [4]. The categorical scoring method was originally developed for the wool-carder bee *Anthidium manicatum* [4], but has been applied to assess wing wear in bumble bees previously [1,3,18]. The method involves assigning qualitative observations of wing wear to seven wing scores of increasing degradation, ranging from intact wings (WW0) to highly worn wings (WW6). Furthermore, two quantitative wing morphometrics were measured. These were perimeter-area ratio and FD of imaged wings which were both measured in Python 3.13.

**Fig 1:**
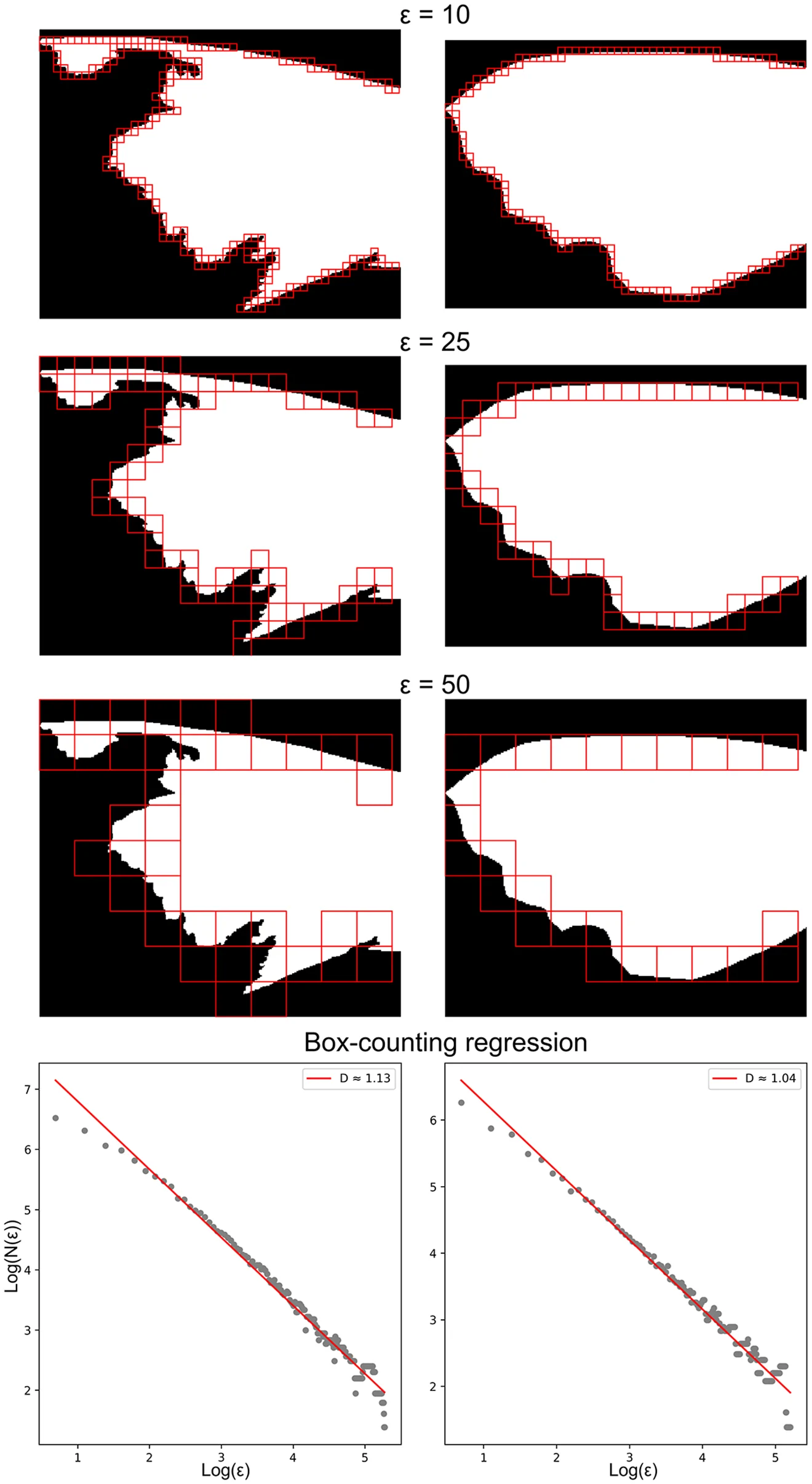
Illustration of the standard box-counting method used to calculate fractal dimension in two bee wings; a heavily worn wing (left; D ≈ 1.13) and a lightly worn wing (right; D ≈ 1.04). The wings are covered with a square grid of uniform box sizes (ε), and the number of boxes intersecting the shape, N(ε), is counted at varying box sizes. The slope of the resulting log-log plot yields the fractal dimension.

**Fig 2:**
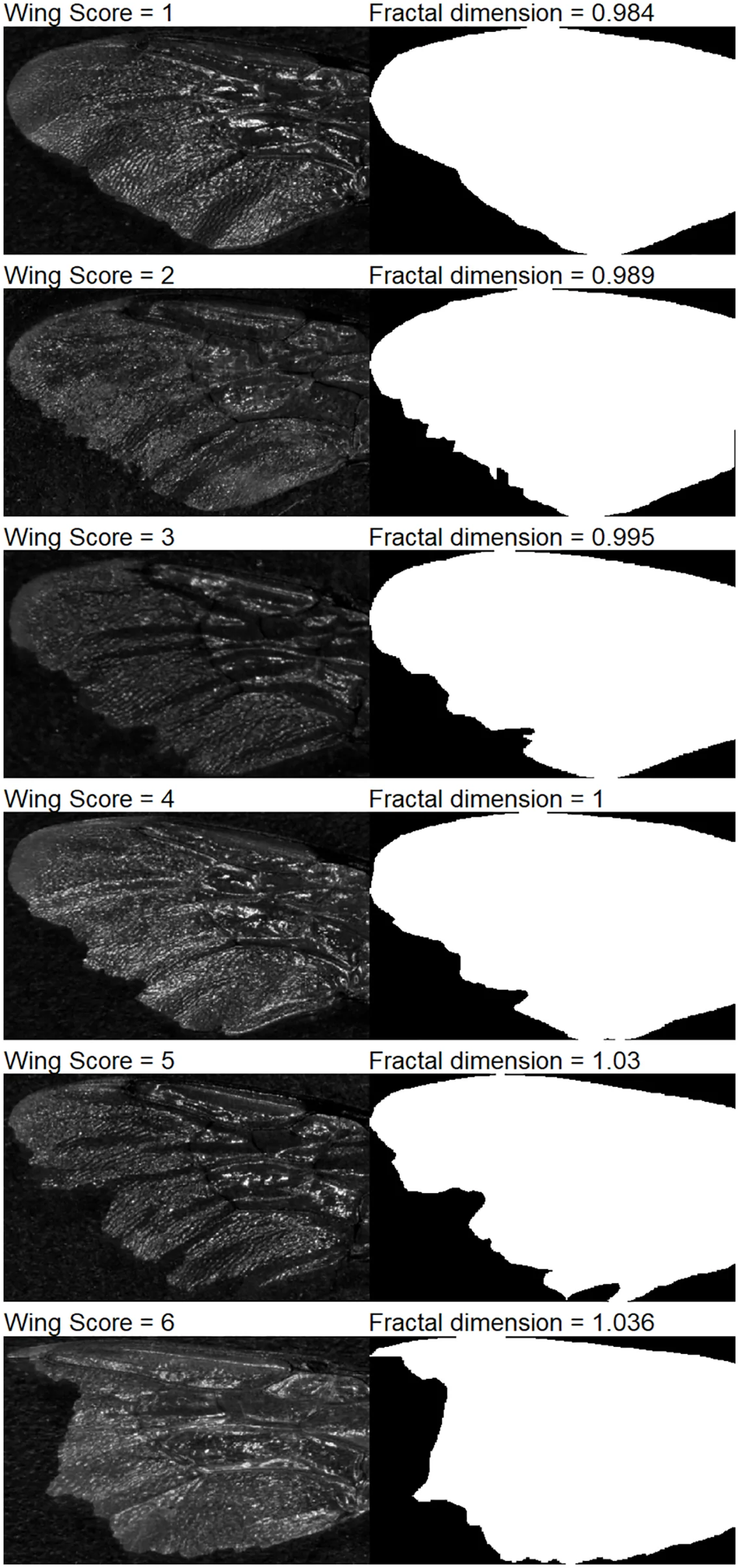
Visual comparison of wing wear scores with corresponding binary masks and computed fractal dimension values. Images illustrate the stages of wing wear (WW0-WW6) adapted from Mueller & Wolf-Mueller [4]; WW0 with the wing margin completely intact, progressively degrading through WW1 with margin showing one or two nicks, WW2 with margin showing 3 to 10 nicks, WW3 with margin almost completely serrated with more than 10 nicks, but some original margin intact, WW4 with margin completely serrated with excisions less than half the width of the distal submarginal cell and no original margin intact, WW5 with margin completely serrated with excisions more than half, but less than the entire width of the distal submarginal cell, culminating with WW6 margin exhibiting complete serration and excisions greater than the width of the distal submarginal cell.


Fractal dimension was measured using the box-counting method [19–21] (Fig 1 and 2). Prior to analysis, all binary masks were standardized to a uniform width of 512 pixels to ensure consistent spatial scale across specimens. To calculate FD using the box-counting method, a grid of square boxes of side length ε is placed over the binary mask and the number of boxes N(ε) that intersect the wing margin is counted. This process is repeated across progressively larger box sizes, ranging from ε = 2 pixels to half the minimum image dimension. The FD is calculated as:

$$\begin{array}{c} D=\underset{\varepsilon \to \text{0}}{\text{lim}}\frac{\text{log }N(\varepsilon )}{-\text{log}\varepsilon } \end{array}$$

where *D* is estimated as the absolute slope of the relationship between the logarithm of the number of boxes required to cover the wing margin and the logarithm of the box size. A higher FD indicates a more complex and fragmented wing margin, while a lower value indicates a smoother one.

### Statistical analysis

All statistical analyses were completed in R v4.2.2 [22]. The FD of undamaged wings (*i.e.,* wings with a visually assessed wing score of 0) were compared between species with an ANOVA and then followed with the Tukey post-hoc test using the emmeans package [23] to determine between species differences in FD. The ANOVA model fit was evaluated through inspection of the model residuals using a Q-Q plot (S2 Fig). Following this, the Pearson correlation coefficient was calculated and a t-test completed to determine the magnitude and significance of the correlation between wing wear score and perimeter-area ratio or FD. To determine whether body size influences wing degradation, Pearson coefficients were computed to test the correlation between ITD and perimeter-area ratio or FD.

## Results

**Fig 3:**
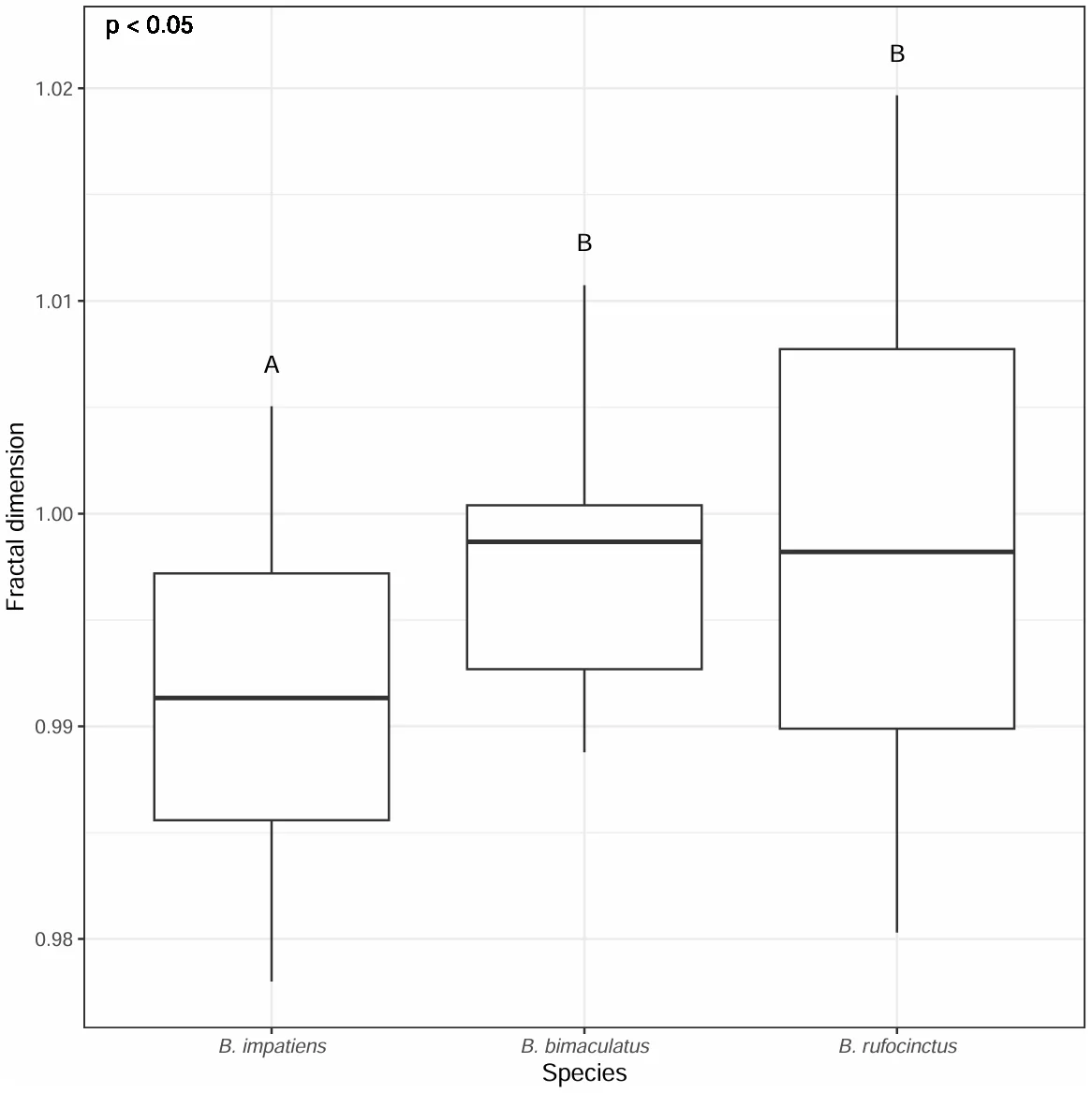
Comparison of fractal dimension (wing score = 0) between undamaged wings (wing wear score = 0) of *Bombus impatiens*, *B. rufocinctus* and *B. bimaculatus.* Letters indicate significant differences between groups according to post hoc analysis.

In total, 263 bumble bees were collected across both field seasons. A total of 109 *Bombus impatiens* (2016 = 18, 2017 = 91), 82 *B. bimaculatus* (2016 = 26, 2017 = 56) and 72 *B. rufocinctus* (2016 = 51, 2017 = 21). The FD of undamaged wings of *B. impatiens* was on average 0.991, *B. bimaculatus* 0.998 and *B. rufocinctus* 0.998. The difference between *B. impatiens* and the two other species was statistically significant according to the computed ANOVA (Fig 3; F = 3.71, p = 0.03).

**Fig 4:**
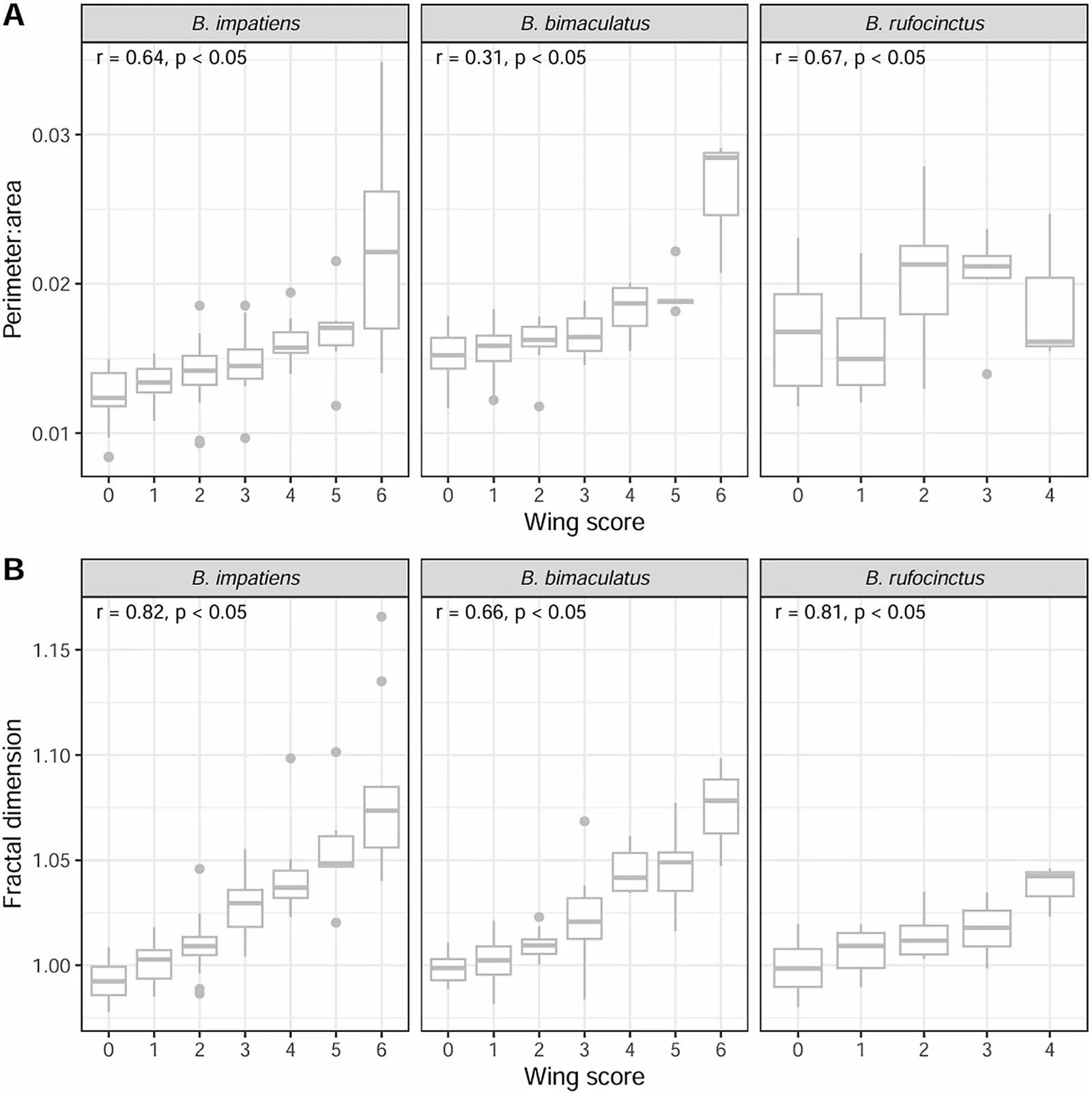
Correlation between wing wear score and (A) perimeter-area ratio and (B) fractal dimension by species. The magnitude (*r*) and significance (*p*) of the Pearson product correlation are displayed on the top-left of each plot.

Wing wear score was more strongly correlated with fractal dimension (*B. impatiens*: *r* = 0.82, *p* < 0.01; *B. bimaculatus*: r = 0.81, p < 0.01; *B. rufocinctus*: *r* = 0.66, *p* < 0.01) than perimeter-area ratio (*B. impatiens*: *r* = 0.64, *p* < 0.01; *B. bimaculatus*: r = 0.67; p < 0.01; *B. rufocinctus*: *r* = 0.31, *p* = 0.01) for all three bumble bee species (Fig 4).

**Fig 5:**
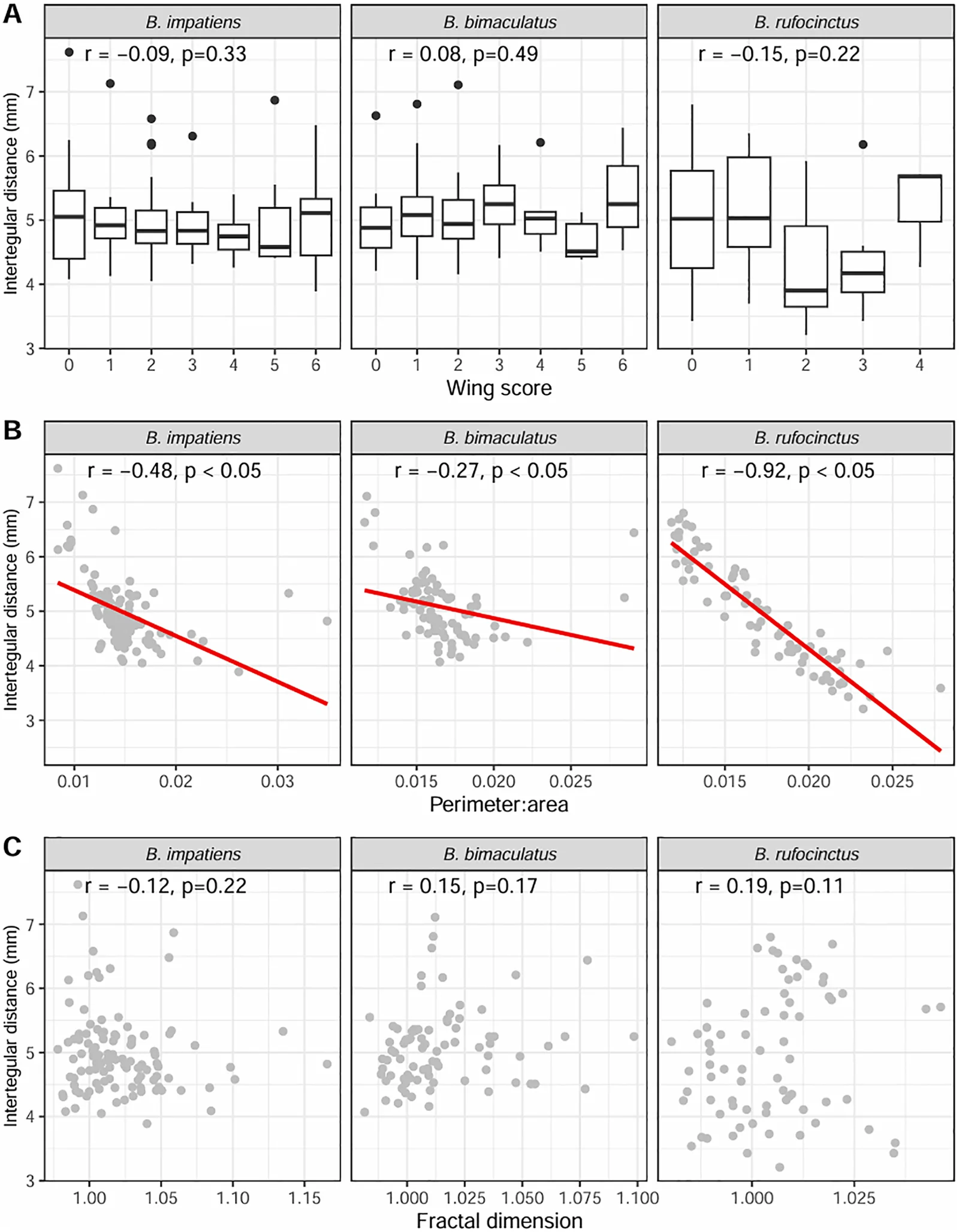
Correlation between intertegular distance and (A) wing wear score, (B) perimeter-area ratio and (C) fractal dimension. The fitted red line is estimated from a simple regression.

Intertegular distance (ITD) was uncorrelated with wing wear score (Fig 5; *B. impatiens*: *r* = −0.09, *p* = 0.33; *B. bimaculatus*: *r* = 0.08, p = 0.49; *B. rufocinctus*: *r* = −0.15, *p* = 0.22) and FD (*B. impatiens*: *r* = −0.12, *p* = 0.22; *B. bimaculatus*: *r* = 0.15, p = 0.17; *B. rufocinctus*: *r* = 0.19, *p* = 0.11). Conversely, perimeter-area ratio was strongly negatively correlated with ITD for all species (*B. impatiens*: *r* = −0.48, *p* < 0.01; *B. bimaculatus*: r = −0.27; p = 0.01; *B. rufocinctus*: *r* = −0.92, *p* < 0.01).

## Discussion

We found that FD varies between undamaged wings of three eastern North American bumble bee species and therefore might be a wing morphometric that varies interspecifically among bumble bees. The individual variation in FD reflects the natural developmental variation in wing morphology documented in bumble bees [24].

Among bees with damaged wings, we show that FD is a promising morphometric for assessing wing degradation. FD showed a stronger correlation with MWM scores than the commonly used perimeter-area ratio, suggesting that FD may capture subtle wing degradations not detectable by traditional metrics. This aligns with Cartar [3] and Foster & Cartar [1] who demonstrated progressive wing wear with foraging effort and supports FD as a quantitative complement to categorical scoring systems such as that developed in Mueller & Wolf-Mueller [4].

Contrary to our hypothesis relating wing wear to body size, we found that neither MWM score nor wing fractal dimension were significantly correlated with bumble bee body size. The lack of correlation was unexpected given that wing wear has been found to primarily result from collisions with vegetation, and collision mechanics would predict greater impact forces for larger-bodied bees [1]. Several factors might explain this absence of a detectable intraspecific relationship. Within-colony behavioural partitioning may decouple body size from foraging intensity as larger workers tend to forage over greater distances on dispersed resources while smaller workers may forage locally in denser vegetation [13,18]. Furthermore, compensatory flight mechanics such as differences in speed and maneuverability between size classes may alter collision frequencies in ways that offset the mass-related differences in impact force [1,25].

The correlation of perimeter-area ratio with body size was negative which opposed our prediction. Although body size was uncorrelated with the MWM score, smaller bees may experience greater wing wear due to a multitude of factors; higher wing loading, greater wingbeat frequencies and more frequent maneuvering through dense floral patches may increase the total accumulation of wing wear [18,26]. Increased wing damage in these smaller individuals could in turn limit their ability to travel long distances or move in complex environments [27], making them more dependent on local floral resources.

We note that while ITD is widely used as a body size proxy, recent work has shown that its relationship with dry mass varies at the intraspecific level and may be inconsistent across bee taxa [28]. Within *Bombus* specifically, the ITD-dry mass relationship appears more reliable than in other genera, but the absence of a detectable body size effect on wing wear in our study should still be interpreted with caution as ITD may not fully capture the variation in body mass that might influence foraging behaviour and wear accumulated [28].

Furthermore, the relationship between wing wear and body size is confounded with age-related wear in our dataset. Bumble bee colonies produce workers continuously throughout the season, and it is therefore likely that our cross-sectional samples contained a mixture of old and young workers with correspondingly different foraging experience. This confounding of age-related wear is inherent to field studies of wing wear and cannot be fully resolved without the marking and re-capture of individual workers over time [3]. However, Foster and Cartar [1] demonstrated that wing wear in bumble bees results primarily from wing collisions with vegetation during foraging rather than from flight time, suggesting that wear reflects foraging behaviour, not chronological age. Future studies seeking to elucidate the mechanisms behind wing wear should use multiple direct measures of body mass and incorporate marked individuals of known age to disentangle these effects.

Bumble bee movement can be directly monitored using radio-frequency identification (RFID) to record when individually tagged workers leave and return from an experimentally deployed colony [15]. While RFID technology allows direct quantification of foraging effort, this approach is not without limitations. Specifically, the time, cost, and expertise involved in the deployment of experimental colonies, as well as the restriction of the approach to large-bodied insects such as bumble bees that are not impeded in movement by RFID tags. In field studies where individual foraging insects are not tracked over time, wing wear can be used as a proxy for foraging effort [5,6]. This is applicable to the in-situ monitoring of wild bees, a situation where the locations of nests are likely to be unknown and where body size varies interspecifically. For example, Chau et al. [6], recently used categorical wing wear scores as a proxy for foraging effort to infer the relationship between urbanization and foraging effort, while Borchardt et al. [5] measured wing area to investigate the impact of prairie restoration on wild bee foraging effort.

Our findings suggest that fractal dimension analysis provides a sensitive, quantitative alternative to current wing wear survey methods. FD provides continuous measurements sensitive to the subtle serrations of worn wings, and the strong correlation between FD and the widely used Mueller and Wolf-Mueller method validates its potential use as a more specific proxy. Its quantitative nature also enables standardized measurements across studies. As a metric that can be readily calculated from wing photographs, FD offers a practical tool for assessing bee body condition in sites where individual tracking may not be feasible. Future studies investigating the use of FD measurements to infer bumble bee colony-level performance or responses to environmental stressors could be useful for further advancing pollinator health monitoring.

## Supporting information

S1 Fig Location of meadow field sites at Rouge National Urban Park. The green polygons depict the boundary of each surveyed habitat patch and the black points show the location of vegetation surveys.
(TIFF)

S2 Fig Q-Q plot of model residuals for ANOVA of wing fractal dimension (FD) on intertegular distance. (PNG)
